# Supplementary figures and images for: Association between prior tuberculosis disease and dysglycemia within an HIV-endemic, rural South African population
Source: PLoS One. 2023 Mar 16;18(3):e0282371. doi: 10.1371/journal.pone.0282371 (PMC10019670; doi:10.1371/journal.pone.0282371)

**S1 Fig. Analytic Cohort Flow Diagram**

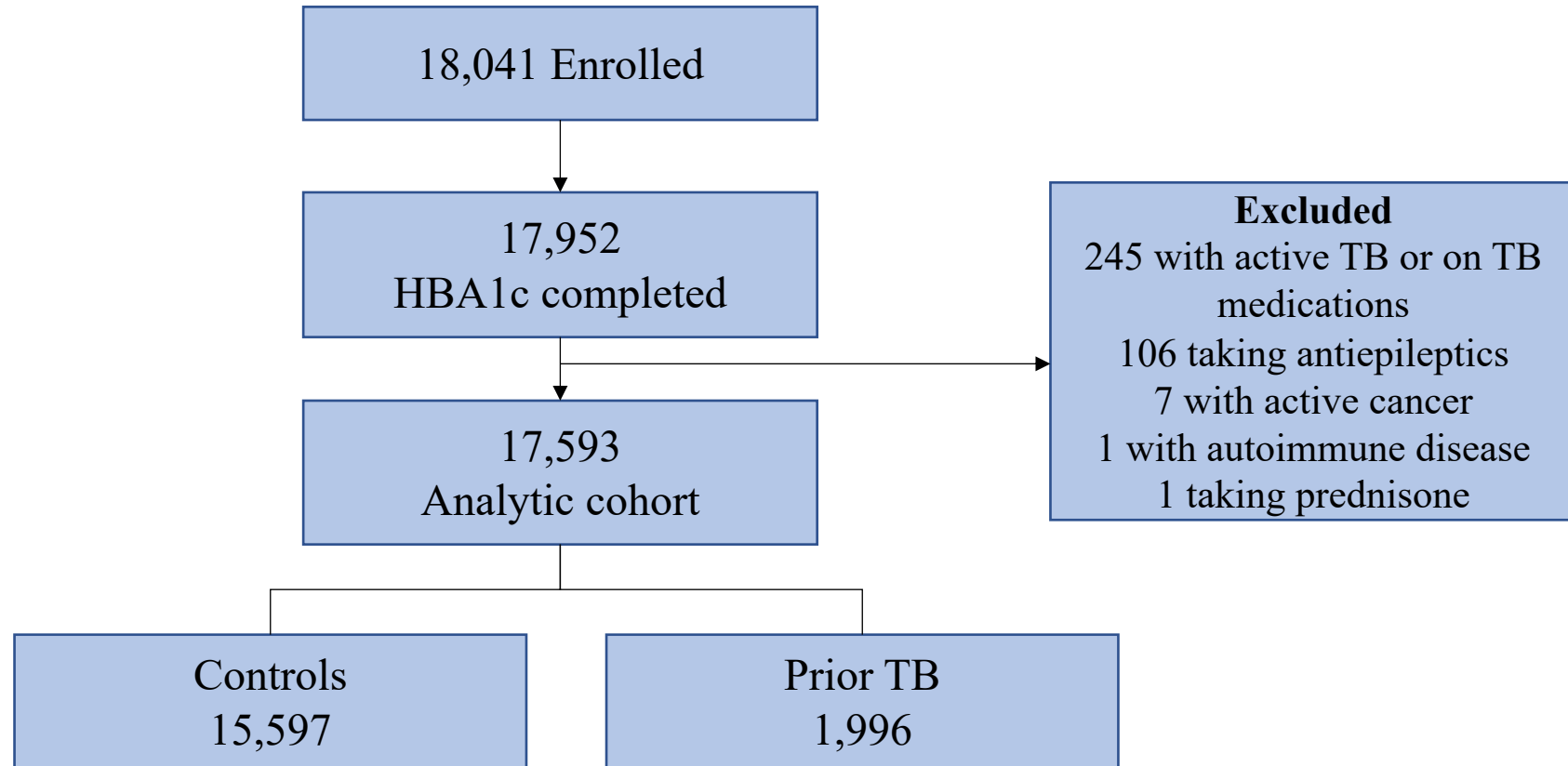

Supplement: S1 Fig — (PDF) [file pone.0282371.s001.pdf]

S2 Fig. Sex-stratified original cohorts versus matched cohorts based on age

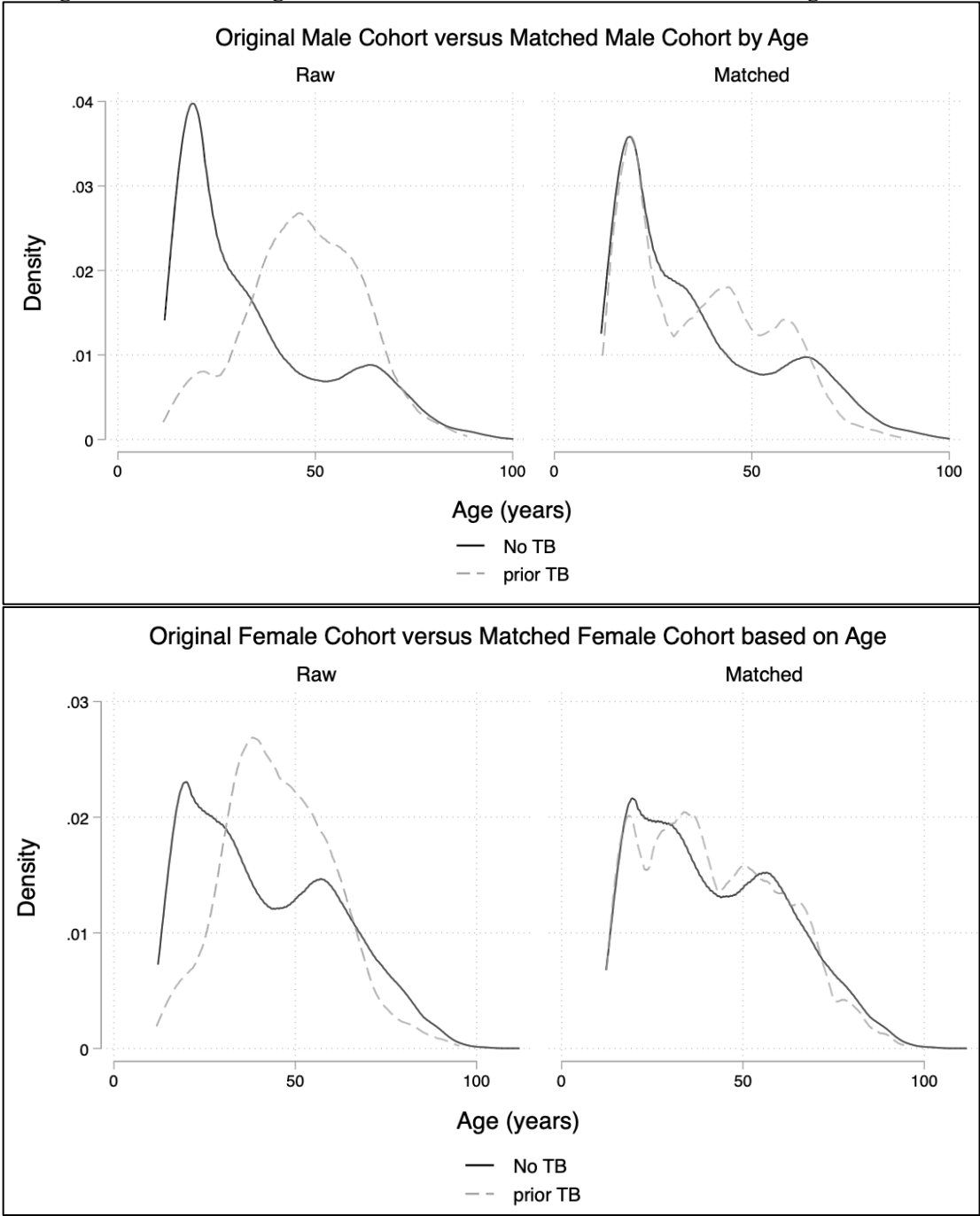

Supplement: S2 Fig — (PDF) [file pone.0282371.s002.pdf]
