# Supplementary material for: Association between prior tuberculosis disease and dysglycemia within an HIV-endemic, rural South African population
Source: PLoS One. 2023 Mar 16;18(3):e0282371. doi: 10.1371/journal.pone.0282371 (PMC10019670; doi:10.1371/journal.pone.0282371)
Supplement: S1 Table — (PDF) [file pone.0282371.s003.pdf]

Table S1: Baseline cohort characteristics by sex and prior TB status

Characteristic	Males		Females	
	**Controls** n=4,847	**Prior TB** n=760	**Controls** n=10,750	**Prior TB** n=1,236
Age (year)	34.3 ± 18.8	47.0 ± 14.4	42.2 ± 19.8	45.6 ± 14.6
HIV positive (%)	18.5	60.3	34.0	75.7
Waist Circumference (cm)	79.6 ± 12.4	81.7 ± 11.2	91.3 ± 17.2	90.2 ± 15.5
Socioeconomic Score*	0.3 ± 2.0	-0.04 ± 2.1	0.24 ± 2.0	0.04 ± 1.9
Past or Active Smoker (%)	19.5	37.4	1.2	2.6
Consumes Alcohol (%)	26.8	41.1	5.2	8.0

Socioeconomic Score* ranges from -7.0 to +7.0
