# Supplementary material for: Association between prior tuberculosis disease and dysglycemia within an HIV-endemic, rural South African population
Source: PLoS One. 2023 Mar 16;18(3):e0282371. doi: 10.1371/journal.pone.0282371 (PMC10019670; doi:10.1371/journal.pone.0282371)
Supplement: S2 Table — (PDF) [file pone.0282371.s004.pdf]

Table S2: Standardized differences between raw and matched cohort

Characteristic	Standardized Differences		Variance Ratio	
	Raw Cohort	Matched Cohort	Raw Cohort	Matched Cohort
Males				
Age	0.7512394	0.0348265	0.5867181	0.7852276
HIV positive	0.9532535	0.0104244	1.604853	1.01286
Waist Circumference	0.1839929	-0.0608697	0.8184932	0.9984563
Socioeconomic Score*	-0.1699725	0.0506393	1.169031	0.9137868
Smoker (%)	0.4067466	-0.0429801	1.498389	0.9413861
Consumes Alcohol (%)	0.3030623	-0.1099956	1.232288	0.8913289
Females				
Age	0.1960013	-0.0142081	0.5517675	0.8954692
HIV positive	0.9228421	0.002245	0.8189807	1.001068
Waist Circumference	-0.0665615	-0.2851283	0.8187331	0.8105264
Socioeconomic Score*	-0.1065839	-0.0284168	0.9298806	0.8470229
Smoker (%)	0.0994357	0.0070238	2.067242	1.060343
Consumes Alcohol (%)	0.1142944	0.0095237	1.501108	1.037597

Socioeconomic Score* ranges from -7.0 to +7.0 with a mean score of 0.45 (standard deviation of 2.0). Standardized differences of <0.25 and variance ratios between 0.5-2.0 indicate good balance between the matched samples.
