# Supplementary material for: Association between prior tuberculosis disease and dysglycemia within an HIV-endemic, rural South African population
Source: PLoS One. 2023 Mar 16;18(3):e0282371. doi: 10.1371/journal.pone.0282371 (PMC10019670; doi:10.1371/journal.pone.0282371)
Supplement: S3 Table — (PDF) [file pone.0282371.s005.pdf]

Table S3. Weight applied to each matched control

Weight	Male Controls		Female Controls	
	Persons	Percent	Persons	Percent
1	422	78.00	853	86.42
2	95	17.56	113	11.45
3	15	2.77	17	1.72
4	5	0.92	4	0.41
5	3	0.55	0	0
6	1	0.18	0	0
Total matched persons	541	100.00	987	100.00

Each control person is matched with one or more persons with prior TB. All single matches have a weight=1, whereas a control person matched to 3 persons with prior TB would have a weight=3.
