# Supplementary material for: Association between prior tuberculosis disease and dysglycemia within an HIV-endemic, rural South African population
Source: PLoS One. 2023 Mar 16;18(3):e0282371. doi: 10.1371/journal.pone.0282371 (PMC10019670; doi:10.1371/journal.pone.0282371)
Supplement: Attachment — Submitted filename: Response to Reviewers.pdf [file pone.0282371.s007.pdf]

Dr. Elizabeth S. Mayne
Academic Editor
PLOS ONE

2nd December 2022

Dear Dr. Mayne and editorial team,

Thank you for inviting us to submit a revised draft of our manuscript entitled "*Association between prior tuberculosis disease and dysglycemia within an HIV-endemic, rural South African population*" to PLOS ONE. We appreciate the time and effort you and each reviewer have dedicated to providing insightful feedback to strengthen our paper. We followed the reviewers' suggestions and have outlined the revisions to the paper below. We hope that our edits and the responses we provide below satisfactorily address all the issues and concerns you and the reviewers have noted.

JOURNAL REQUIREMENTS:

1. Please ensure that your manuscript meets PLOS ONE's style requirements, including those for file naming.

RESPONSE: The title page, abstract, and manuscript have been edited with tracked changes to meet all of the style requirements instructed above.

2. Please include a complete copy of PLOS' questionnaire on inclusivity in global research in your revised manuscript.

RESPONSE: A section on Inclusivity in global research is now included in our methods section, and a complete questionnaire is attached as a supplement (S4 Checklist).

3. In your data availability statement, you have not specified where the minimal data set underlying the results described in your manuscript can be found. PLOS defines a study's minimal data set as the underlying data used to reach the conclusions drawn in the manuscript and any additional data required to replicate the reported study findings in their entirety. All PLOS journals require that the minimal data set be made fully available. Upon re-submitting your revised manuscript, please upload your study's minimal underlying data set as either Supporting Information files or to a stable, public repository and include the relevant URLs, DOIs, or accession numbers within your revised cover letter. For a list of acceptable repositories, please see http://journals.plos.org/plosone/s/data-availability#loc-recommended-repositories. Any potentially identifying patient information must be fully anonymized.

RESPONSE: Our current data statement is updated to include the following bolded words: "Data and the data dictionary defining each field **can be accessed at https://data.ahri.org/index.php/catalog/1006** via the Africa Health Research Institute Data Repository**.** Please email RDMServiceDesk@ahri.org. **Access can be granted** after publication

and upon approval of the proposed analyses by the Vukuzazi Scientific Steering Committee and completion of a data access agreement."

4. One of the noted authors is a group or consortium the Vukuzazi Study Team. In addition to naming the author group, please list the individual authors and affiliations within this group in the acknowledgments section of your manuscript. Please also indicate clearly a lead author for this group along with a contact email address.

RESPONSE: The individual authors and their affiliations within the Vukuzazi Study Team are now listed within the Acknowledgments section. Emily Wong is the lead author for the Vukuzazi Study Team and may be contacted at Emily.wong@ahri.org.

5. Please review your reference list to ensure that it is complete and correct. If you have cited papers that have been retracted, please include the rationale for doing so in the manuscript text or remove these references and replace them with relevant current references. Any changes to the reference list should be mentioned in the rebuttal letter that accompanies your revised manuscript. If you need to cite a retracted article, indicate the article's retracted status in the References list and also include a citation and full reference for the retraction notice.

RESPONSE: All references were reviewed, and no papers have been retracted.

REVIEWER #1 COMMENTS:
Thank you for your time and effort in giving us feedback. We address your comments one by one in the following.

1. Approximately half of the eligible residents in the DHSS participated in the study. It would be useful to know the basic characteristics of those who did not take part, so the reader can make an assessment of the generalisability of the results

RESPONSE: Lines 198-200 now include the basic demographic information (sex and age) of the eligible participants who did not enroll in the Vukuzazi Study. We state, "Of the eligible participants within AHRI's DHSS, women (OR 2.03 [95% CI 1.94-2.12]; $p<0.001$) and individuals over the age of 50 (OR 2.01 [95% CI 1.92-2.11]; $p<0.001$) were more likely to attend the Vukuzazi Study compared to men and younger participants."

2. The mean age of the study population is rather low and the background prevalence of diabetes would also be expected to be fairly low. The authors should comment on this limitation.

RESPONSE: Whereas the study population is representative of the rural region where our study was conducted, we acknowledge that the age distribution of this profile is younger than in high-income countries and therefore resulted in a relatively lower prevalence of diabetes. The following statement is now added to lines 271-273. "Reflecting the population of rural South

Africa, the mean age of our cohort was relatively young (men 36.2 years; women 42.5 years) compared to high-income settings [44]. This also likely resulted in a comparatively lower prevalence of dysglycemia."

3. Reference 1 is only cited after several other references-this should be corrected.

RESPONSE: This was an error with EndNote that has now been resolved.

4. The word "comprise" is not followed by "of"

RESPONSE: Line 202. The statement "was comprised of" was changed to "included".

5. The references should be formatted in a standardised fashion.

RESPONSE: References are organized using EndNote using the PLoS style format.

REVIEWER 2 COMMENTS:

1. There are a number of African studies that have shown that the association of TB and diabetes varies with the diagnostic test used. This may have relevance for your finding 1. Eur Respir J. 2017 Jul; 50(1): 1700004. 2.. J Infect Dis 2016; 213: 1163–1172. 3.Int J Tuberc Lung Dis 2017 Feb 1;21(2):208-213.

RESPONSE: We appreciate the reviewer bringing these studies to our attention. The diagnostic tests used in all three of these studies were used to detect acute TB infection. By contrast, considered the effect of previously treated TB and dysglycemia. Nonetheless, these studies raise important questions about the validity of hemoglobin A1c testing for diagnosing diabetes in key populations and we have incorporated two of them into lines 305-307.

Thank you for giving us the opportunity to strengthen our manuscript with your valuable comments and queries. We've incorporated your feedback and hope the revised manuscript will be suitable for publication in PLOS ONE.

Sincerely,

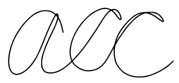

Alison Castle
Corresponding Author
Africa Health Research Institute
719 Umbilo Rd, Durban, Kwa-Zulu Natal, 4001
Alison.castle@ahri.org
