# Supplementary material for: Association between prior tuberculosis disease and dysglycemia within an HIV-endemic, rural South African population
Source: PLoS One. 2023 Mar 16;18(3):e0282371. doi: 10.1371/journal.pone.0282371 (PMC10019670; doi:10.1371/journal.pone.0282371)
Supplement: Attachment — Submitted filename: Rebuttal Letter2_Jan2023.pdf [file pone.0282371.s008.pdf]

Dr. Elizabeth S. Mayne
Academic Editor
PLOS ONE

20th January 2023

Dear Dr. Mayne and editorial team,

Thank you for inviting us to submit a revised draft of our manuscript entitled "*Association between prior tuberculosis disease and dysglycemia within an HIV-endemic, rural South African population*" to PLOS ONE. We followed the reviewer's suggestions and have outlined the revisions to the paper below. We hope that our edits and the responses we provide below satisfactorily address all the issues and concerns you and the reviewers have noted.

REVIEWER #1 COMMENTS:
My only additional comment regards the formatting of references. The authors should be consistent in the use of upper case letters in the titles of their references. In some instances sentence case is used while in others, every word in the title begins with an upper case letter.

RESPONSE:

We appreciate you highlighting the discrepancies in our reference titles. The following references have now been reformatted so that all are in sentence case only: 5, 8, 11, 12, 15, 16, 17, 20, 21, 22, 23, 25, 28, 30, 31, 32, 33, 34.

Thank you for giving us the opportunity to strengthen our manuscript with your valuable comments. We've incorporated your feedback in the revised manuscript and hope this will be suitable for publication in PLOS ONE.

Sincerely,

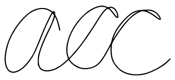

Alison Castle
Corresponding Author
Africa Health Research Institute
719 Umbilo Rd, Durban, Kwa-Zulu Natal, 4001
Alison.castle@ahri.org
